# Microencapsulated Hepatocytes Differentiated from Human Induced Pluripotent Stem Cells: Optimizing 3D Culture for Tissue Engineering Applications

**DOI:** 10.3390/cells12060865

**Published:** 2023-03-10

**Authors:** Marwa Hussein, Mattia Pasqua, Ulysse Pereira, Nassima Benzoubir, Jean-Charles Duclos-Vallée, Anne Dubart-Kupperschmitt, Cecile Legallais, Antonietta Messina

**Affiliations:** 1 UMR_S 1193, INSERM/Université Paris Saclay, F-94800 Villejuif, France; 2 Fédération Hospitalo-Universitaire (FHU) Hépatinov, F-94800 Villejuif, France; 3 UMR CNRS 7338 Biomechanics & Bioengineering, Université de Technologie de Compiègne, Sorbonne Universités, F-60203 Compiegne, France

**Keywords:** hiPSCs, hiPSC-derived hepatocytes, alginate microencapsulation, 3D culture conditions, hepatocyte functions, hepatocyte maturation, encapsulated iHep organoids

## Abstract

Liver cell therapy and in vitro models require functional human hepatocytes, the sources of which are considerably limited. Human induced pluripotent stem cells (hiPSCs) represent a promising and unlimited source of differentiated human hepatocytes. However, when obtained in two-dimensional (2D) cultures these hepatocytes are not fully mature and functional. As three-dimensional culture conditions offer advantageous strategies for differentiation, we describe here a combination of three-dimensional (3D) approaches enabling the successful differentiation of functional hepatocytes from hiPSCs by the encapsulation of hiPSC-derived hepatoblasts in alginate beads of preformed aggregates. The resulting encapsulated and differentiated hepatocytes (E-iHep-Orgs) displayed a high level of albumin synthesis associated with the disappearance of α-fetoprotein (AFP) synthesis, thus demonstrating that the E-iHep-Orgs had reached a high level of maturation, similar to that of adult hepatocytes. Gene expression analysis by RT-PCR and immunofluorescence confirmed this maturation. Further functional assessments demonstrated their enzymatic activities, including lactate and ammonia detoxification, as well as biotransformation activities of Phase I and Phase II enzymes. This study provides proof of concept regarding the benefits of combining three-dimensional techniques (guided aggregation and microencapsulation) with liver differentiation protocols as a robust approach to generate mature and functional hepatocytes that offer a permanent and unlimited source of hepatocytes. Based on these encouraging results, our combined conditions to produce mature hepatocytes from hiPSCs could be extended to liver tissue engineering and bioartificial liver (BAL) applications at the human scale for which large biomasses are mandatory.

## 1. Introduction

Liver failure is a devastating condition of varied origin that can lead to progressive multi-organ failure resulting from disruption of the metabolic activities of the liver, the organ largely responsible for maintaining homeostasis in the body. While orthotopic liver transplantation (OLT) is the only effective solution to halt acute or fulminant liver failure, it is limited by the shortage of transplantable organs available to meet demand. Further, the indications for OLT are broadening, thus increasing the gap between need and donor organ availability [1]. There has therefore been much interest in developing strategies that might serve as substitutes to ensure liver function for patients awaiting transplantation and thus act as a bridge until OLT is possible. These include cell therapy approaches in the form of bioengineered liver tissues and extracorporeal bioartificial liver devices (BALs) [2].

Because biosynthesis and enzymatic detoxification in the liver are assured by highly functional hepatocytes, a metabolically active hepatocyte biomass is necessary for any liver cell therapy approach. Primary human hepatocytes (PHHs) are the most obvious candidates, as they ensure in vivo liver functionality [3]. Nevertheless, their short life span, in vitro phenotypic instability and variable quality (dependence on organ health status, inter-subject/donor variability), as well as the cell damage caused by cryopreservation, significantly limit their use [4]. Alternatives have therefore long been considered. Primary porcine hepatocytes and cancer-derived hepatocyte cell lines (HepG2/C3A, HuH7, HepaRG) have been suggested or even evaluated in preclinical/clinical trials, but neither of these possibilities can hold up in clinical applications because of the risk of zoonosis or of their limited liver functions, respectively [5]. The advent and therapeutic potential of human pluripotent stem cells (hPSCs) hold great promise for both liver regenerative medicine and tissue engineering. In theory, the generation of differentiated hepatocytes from hPSCs would bypass the shortage of liver donors, as well as avoiding the aforementioned safety issues [6]. The generation of new hepatocyte sources from hPSCs has been widely investigated, namely, using human embryonic stem cells (hESCs) and human induced pluripotent stem cells (hiPSCs) applying protocols inspired from embryonic development [7,8,9,10,11]. The resulting cells may express hepatocyte-specific protein markers including albumin (ALB), hepatocyte nuclear factor 4α (HNF4α), asialoglycoprotein receptor (ASGR), α1-antitrypsin (A1AT) and cytochrome P450 (CYP450) isoforms and display hepatocyte functions such as glycogen and lipid storage capacity as well as detoxification and xenobiotic biotransformation activities [11,12].

However, these differentiation protocols have so far failed to enable the full differentiation of hiPSC-derived cells; thus, the production of immature hepatocytes whose phenotype and function were more similar to fetal or neonate hepatocytes than to adult hepatocytes occurred [13]. In this context, efforts to optimize differentiation have been based on previous experience with the culture and maintenance of PHHs in vitro. One strategy that may improve hepatocyte differentiation and functionality is 3D culture, as it both maximizes cell–cell interactions and approximates the physiological environment of the cells [14]. Indeed, 3D culture has been shown to play a major role in hepatocyte maturation when used for the differentiation of hiPSCs [15,16,17]. Cell encapsulation technology in alginate beads is one of the 3D culture methodologies that might represent an interesting tissue engineering approach appropriate for hepatocyte maturation. It consists in trapping the cells within spherical alginate beads that offer protection against shear stress while allowing the diffusion of nutrients and soluble factors through the hydrogel [17]. The structure of this natural and inert biomaterial provides a three-dimensional framework that almost resembles the liver’s mechanical environment [18,19]. Indeed, alginate encapsulation has been applied successfully to the culture and the maintenance of hepatic functions for PHHs and hepatoma cell lines such as HepG2 and HepaRG^TM^ (Biopredic, Rennes, France) [18,20,21]. Furthermore, several studies have demonstrated the beneficial effects of encapsulation on the enhancement of viability and metabolic performance for prolonged periods [22,23]. In addition, alginate beads could easily be cryopreserved and thus rapidly available for use [24,25,26]. However, this technology has not been widely explored in hepatocyte differentiation, and various parameters still need to be studied.

The present study aimed to combine the existing hepatocyte differentiation protocol from hiPSCs with the alginate encapsulation approach as the 3D culture condition and to examine whether we could control hepatocyte differentiation using this combination. We first derived hepatoblasts (iHBs) using a previously published 2D protocol [27]. We then encapsulated iHBs in alginate beads while pursuing direct differentiation into hepatocytes. Because it was possible to modify important parameters during encapsulation, including cell seeding density and culture conditions within the alginate beads, we can present alginate encapsulation as a 3D culture strategy that can achieve hepatocyte differentiation.

## 2. Materials and Methods

### 2.1. Hepatocyte Differentiation from hiPSCs (iHeps)

hiPSCs were differentiated into hepatoblasts (iHBs) according to our previously published protocol [27], which is detailed in the Supplementary Materials and Table S1. At Day 11 of differentiation, the iHBs thus obtained were detached using StemPro Accutase cell dissociation solution (Gibco). Whatever culture system was used, the iHBs were treated according to the differentiation protocol described by Messina et al. [15], using HCM^TM^ Hepatocyte Complete Medium (Lonza, Walkersville, MD, USA) supplemented with 20 ng/mL HGF, 0.1 ng/mL Dexamethasone (Dex) and 20 ng/mL Oncostatin M (OSM), and refreshed every second day until Day 22. On Day 18, in addition to the factors mentioned above, 10 ng/mL vitamin K (VK) (Roche, Basel, Switzerland) was added to the culture medium until the end of differentiation. From Day 21 onwards, additional factors were also added to the medium: 0.5 nM compound E (Santa Cruz Biotechnology, Santa Cruz, CA, USA) and 5 nM SB431542 (Tocris Biosciences, Bristol, UK). From Day 23 to Day 28, the OSM concentration was halved every second day until complete removal while 0.1 and 0.05 ng/mL Dex were added in a cyclical manner to the medium. Details of the growth factors and cytokines used to induce hepatocyte differentiation and maturation are summarized in the Supplementary Materials Table S2.

For 2D culture conditions, which were performed systematically as a control for each differentiation experiment, iHBs were seeded at 2 × 10^5^ cells/cm^2^ density on a homemade coating solution made up of 1% *w*/*v* fibronectin (Sigma-Aldrich, St. Louis, MO, USA), 3% *w*/*v* calf skin collagen type I (Sigma-Aldrich) and 10% *w*/*v* BSA (Sigma-Aldrich). For the 3D culture conditions, iHBs were encapsulated in alginate beads as described in the paragraphs below.

### 2.2. Encapsulation of iHBs as Single Cells in Alginate

Alginate solution was prepared by dissolving alginate powder (Manucol LKX from FMC BioPolymer, Billingstad, Asker 1377, Norway) at 1.5% (*w*/*v*) in a 0.9% (*w*/*v*) NaCl solution. It was sterilized using 0.8, 0.45 and 0.2 µm filters successively. The cell encapsulation protocol was implemented according to a coaxial air flow extrusion method [28]. On Day 11, the iHBs collected were mixed with 1.5% (*w*/*v*) alginate solution to achieve the desired cell density per mL of alginate (4 million, 8 million or 12 million cells/mL alginate). The mixed cell–alginate solution was held in a syringe and extruded through a 24 G nozzle to form droplets, as illustrated in Figure S2. The resulting droplets fell into a gelation bath (NaCl 154 mM, HEPES 10 mM and CaCl_2_ 115 mM, pH 7.4) where they stayed for 15 min to enable alginate polymerization and bead formation. The alginate beads were then washed three times in William’s E medium (WE) and re-suspended in iHep differentiation medium. Empty beads were produced as controls in parallel. The beads were transferred into a culture plate under a rotary orbital shaker (70 rpm). The medium was changed every 2 days according to the protocol described above.

### 2.3. iHB Aggregation Followed by Encapsulation in Alginate

The aggregation of iHBs was achieved via a self-assembly process after their seeding in agarose microwells, as previously reported [15]. An equivalent of 2.5 × 10^5^ iHBs harvested at Day 11 of differentiation were re-suspended in 200 µL HCM and added to each well and then incubated at 37 °C under 5% CO_2_ for 1 h before adding the culture medium supplemented with growth factors. After 24 h of culturing in microwells, the resulting aggregates (4000 cells/aggregate) either underwent a medium change (for the non-encapsulated control condition; iHep-Orgs) or were gently recovered from the agarose microwells and mixed into the alginate solution as described above. An equivalent of 4.5 million seeded iHBs in the form of aggregates were re-suspended in 1 mL 1.5% (*w*/*v*) alginate solution. The steps following the encapsulation of aggregates were the same as those described above for the cells (at the end of the paragraph), allowing self-organization of the aggregates into organoids (Orgs).

### 2.4. Cell Viability Assay

In order to check cell viability following encapsulation and during differentiation, the alginate beads were incubated with 10 μg/mL fluorescein diacetate (FDA) (Sigma-Aldrich) and 1 μg/mL propidium iodide (PI) (Sigma-Aldrich) for 10 min at 37 °C. The beads were then rinsed and imaged using a Leica SP5 confocal microscope.

### 2.5. Assessment of Hepatic Functions In Vitro

#### 2.5.1. Albumin and α-Fetoprotein Synthesis

To quantify albumin (ALB) and α-fetoprotein (AFP) synthesis, the culture medium used for each culture condition was collected 24 h after the medium was refreshed at a specific time point and frozen at −20 °C until analysis. AFP levels were measured using the AFP Human ELISA Kit (Fisher Scientific, Waltham, MA, USA) according to the manufacturer’s instructions. In parallel, albumin secretion was quantified using the human albumin ELISA Quantification Set (Bethyl Laboratories, Montgomery, TX, USA), according to the manufacturer’s description.

#### 2.5.2. Biotransformation Activity—Phase I Metabolism

The activities of Cytochrome P540 1A1/A2 and 3A4 were measured using the 7-ethoxyresorufin-O-deethylase (EROD) assay and Benzyloxyresorufin-O-dealkylase (BROD) assay, respectively. Briefly, beads at either Day 24 or 28 of differentiation were washed with PBS and incubated for 1 h at 37 °C in HBM medium (Lonza) containing 10 μM of substrate; i.e., ethoxyresorufin and 7-benzyloxyresorufin for the EROD and BROD assays, respectively. The substrate solution also included salicylamide (3 mM) and Dicumarol (40 μM) to block Phase II conjugation enzymes. The supernatants were collected and the metabolite (resorufin) was quantified using a fluorescence microplate reader at 595 nm (Spectafluor Plus, TECAN, Männedorf, Switzerland). Further, in order to evaluate the induction of CYP1A1/2 and CYP3A4 enzymes, samples were incubated with either β-Naphthoflavone 100 μM (Sigma-Aldrich) or rifampicin 10 μM (Sigma-Aldrich), respectively, added to the media 72 h before the test.

#### 2.5.3. Biotransformation Activity—Phase II Metabolism

Uridine diphosphate Glucuronosyl Transferase 1A1 activity (UGT1A1) was assessed using an established protocol [29]. Briefly, E-iHep-Orgs were incubated for 1 h at 37 °C with 100 μM 4-methylumbellipherone (4-MU) (Sigma-Aldrich). The supernatants were then collected and stored at −20 °C until analysis. The metabolite was quantified using a fluorescence microplate reader at 450 nm (Spectafluor Plus, TECAN, Männedorf, Switzerland).

#### 2.5.4. Uptake and Release of Indocyanine Green (ICG)

To monitor the uptake and excretion of indocyanine green (ICG), encapsulated iHeps were incubated with 1 mg/mL (5 μM) indocyanine green (Cardiogreen, Sigma-Aldrich) in HCM medium at 37 °C under 5% CO_2_. The ability of the cells to internalize and excrete indocyanine green (ICG) was visualized using phase/contrast microscopy (EVOS™ FL Auto Imaging System, Thermo Fisher Scientific, Waltham, MA, USA) after 1 and 2 h. After 2 h, the ICG solution was removed by washing the encapsulated cells with WE medium 3 times, and the encapsulated iHeps were incubated with HCM culture medium to monitor ICG release.

#### 2.5.5. Lipid Storage—Oil Red O’ Staining

Fixed frozen sections (7 µm) of encapsulated aggregates were treated with 60% isopropanol (Sigma-Aldrich) for 5 min followed by incubation in an Oil Red O Working solution (Sigma-Aldrich) for 10 min. The samples were then washed 5 times with distilled water and counterstained with hematoxylin (Sigma-Aldrich) for 2 min. Finally, the slices were visualized by phase/contrast microscopy (EVOS™ FL Auto Imaging System).

#### 2.5.6. Urea Production and Lactate-Ammonia Detoxification

The ability of cells to detoxify lactate and ammonia at levels higher than physiological norms was assessed after incubating the cells in culture medium supplemented with NH_4_Cl (Sigma-Aldrich) and L-Lactate (Sigma-Aldrich). Two conditions were assessed: (1) the samples were incubated for 2 h with medium containing 1.5 mM NH_4_Cl and 2 mM L-Lactate, as described previously [21], and (2) the samples were incubated for 6 h in ultra-pathological model plasma containing 70 g/L bovine serum albumin (BSA, Sigma-Aldrich), 2 mM NH_4_Cl and 7 mM L-Lactate [30]. After incubation, the supernatant was collected and frozen at −20 °C until analysis. The amounts of detoxified lactate and ammonia were determined by subtracting the amount remaining in the medium. Urea production and lactate and ammonia detoxification in supernatants were analyzed using the QuantiChrom urea assay kit (BioAssay Systems, Hayward, CA, USA), the lactate assay kit (Sigma-Aldrich) and the YDI2950-Indiko™ biochemistry analyzer (Thermo Fisher Scientific), respectively, according to the manufacturers’ instructions.

#### 2.5.7. Data Normalization and Statistical Analysis

Metabolic activities are normalized from the quantity of metabolite produced or consumed/hour/million cells (quantify by DNA extraction as described in the Supplementary Materials). All results were obtained from at least four independent experiments and expressed as mean ± standard deviations. Statistical analysis was performed by one-way ANOVA with the Tukey–Kramer test for multiple comparisons. Values were considered to be significant at *p*-values of <0.0001 (****), <0.001 (***), <0.01 (**) and <0.05 (*).

## 3. Results

### 3.1. iHB Generation and Encapsulation

**Figure 1:**
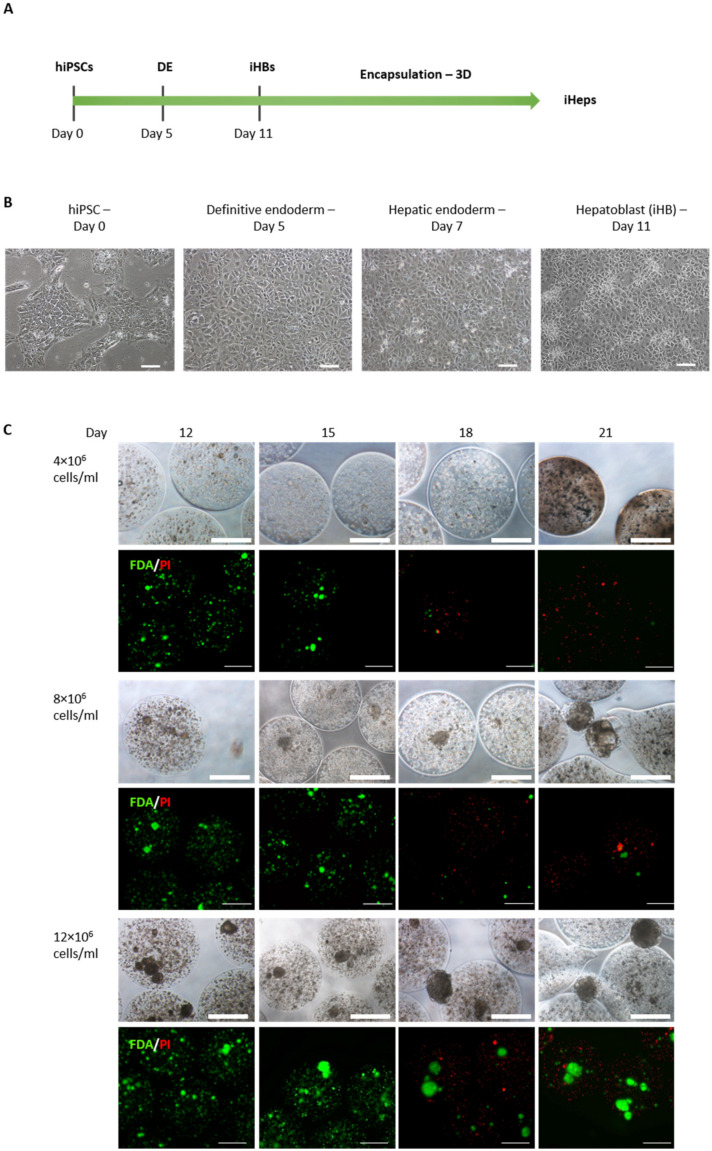
Production of iHeps from hiPSCs and encapsulation as single cells. (**A**) Experimental procedure indicating the course of the differentiation of hiPSCs into hepatoblasts. (**B**) Brightfield images of hiPSC differentiation steps into definitive endoderm (Day 5) and hepatoblasts (Day 11) (scale bar 100 µm). (**C**) Brightfield and fluorescence microscope images of encapsulated iHBs at three cell densities and viability assay during the differentiation procedure. Viable cells are shown in green (fluorescein diacetate—FDA) and dead cells in red (propidium iodide—PI) (scale bar 500 µm).

Figure 1A illustrates the experimental procedures that combined the initial phase of hiPSC differentiation into hepatoblasts and subsequent encapsulation in alginate beads. Hepatoblasts (iHBs) were differentiated under 2D conditions from hiPSCs in successive steps that consisted in the induction of definitive endoderm (DE) until Day 5 and then hepatic specification to generate hepatoblasts (iHBs) at Day 11 (Figure 1B). At Day 5, following treatment with Activin A and LY294002, we obtained a homogeneous cell population that expressed endoderm markers including FOXA2 and GATA4, associated with the disappearance of pluripotent markers expressed in hiPSCs (Supplementary Materials Figure S1A–C). On Day 11, following the addition of further growth factors (BMP4, HGF, FGF4, FGF2), we induced their differentiation towards iHBs that expressed the hepatocyte nuclear transcription factor HNF4α, α-fetoprotein (AFP) and cytokeratin 19 (CK-19). Next, to generate hepatocytes (iHeps) under defined three-dimensional culture conditions, iHBs were harvested and encapsulated in alginate beads, as previously established with HepaRG^TM^ cells [21]. For this purpose, we encapsulated dissociated iHBs in alginate at different cell densities (4, 8 and 12 × 10^6^ cells/mL alginate). After iHB encapsulation, the evolutions of cell morphology and viability were assessed and compared between the three cell densities, as summarized in Figure 1C. Microscopic observations revealed a homogeneous cell distribution within beads approximately 1100 ± 48 µm (*n* = 40) in diameter. The viability of encapsulated iHBs remained high at all cell densities until Day 15 of differentiation (4 days post-encapsulation), with all cells stained green and no dead cells (in red). At the lower cell density (4 × 10^6^ cells/mL), no self-assembly of the cells occurred in the beads. After Day 15, the cells started to die, and all were dead within the next few days. At the higher cell densities (8 and 12 × 10^6^ cells/mL), the cells were able to self-organize within the beads, forming small aggregates after 24 h. These aggregates grew during the period of culturing in beads (Figure 1C). By Day 18 of differentiation, aggregates larger than 250 µm in diameter (45%) escaped from the alginate beads and continued to grow, while those smaller than a diameter of 250 µm remained trapped in the beads. However, only aggregated cells remained viable (stained in green), while the isolated cells were dead (in red). Taken together, these observations indicate that encapsulating iHBs as isolated single cells is not suitable to preserve cell viability and establish 3D culture conditions for efficient hepatocyte differentiation in alginate beads. By contrast, these conditions showed that aggregates could preserve cell viability in alginate beads.

### 3.2. Cell Aggregation of iHBs Prior to Alginate Encapsulation

**Figure 2:**
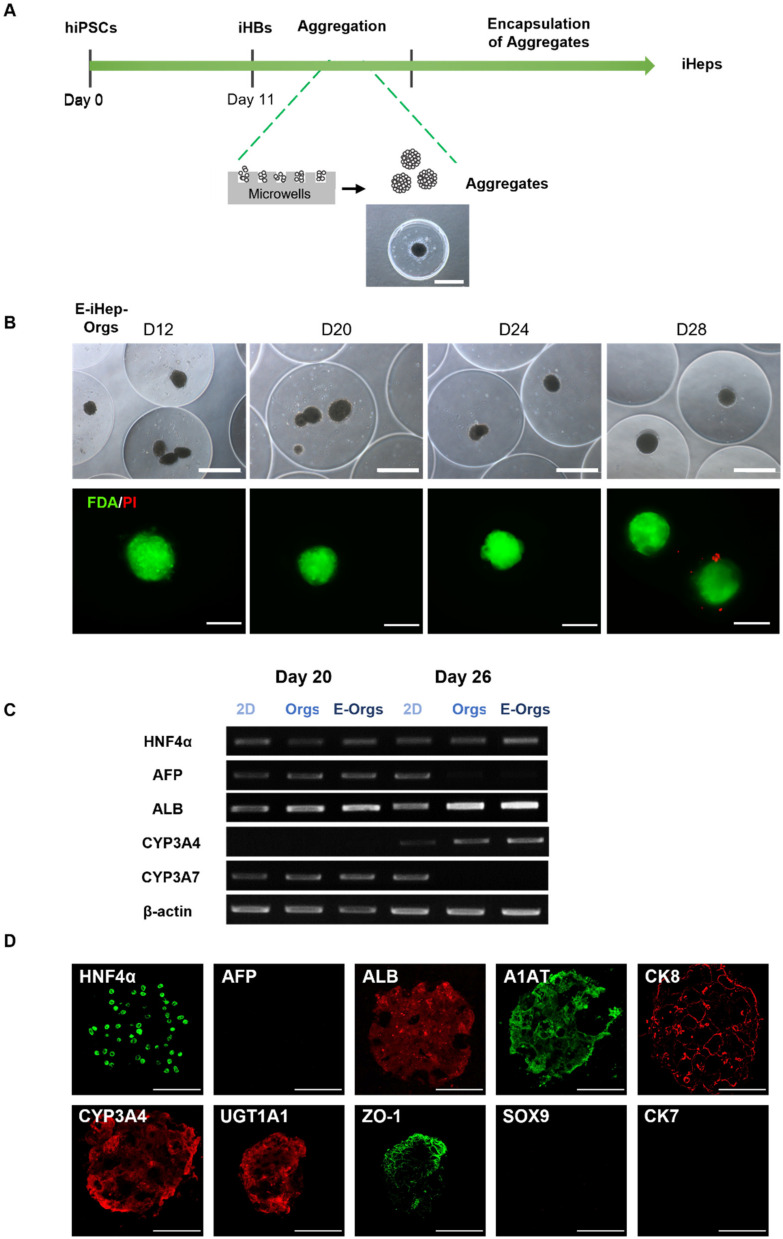
Production of iHeps from iHBs in encapsulated organoids (E-iHep-Orgs). (**A**) Experimental procedure showing the experimental course of differentiation of hiPSCs into functional iHeps. After the differentiation of iHBs from hiPSCs in a monolayer, suspended iHBs were allowed to self-aggregate in microwells and were then encapsulated in alginate beads for differentiation into iHeps (E-iHeps-Orgs). (**B**) Phase contrast microscopy images (scale bar: 500 µm) and assessment of the viability of encapsulated aggregates throughout differentiation under epifluorescence microscopy. In green (fluorescein diacetate—FDA): viable cells. In red (propidium iodide—PI): dead cells (scale bar: 200 µm). (**C**) RT-PCR analysis of HNFα, AFP, ALB, CYP3A4, CYP3A7 in 2D differentiated iHeps, in organoids (Orgs) and in encapsulated organoids (E-Orgs). (**D**) Immunofluorescence staining of E-iHep-Orgs at the final stage of differentiation (Day 28) (scale bar 150 µm).

We next wanted to determine whether aggregation could promote the preservation of cell viability and subsequent differentiation into hepatocytes within alginate beads. To achieve this, we performed the aggregation of iHBs as previously described by Messina et al., 2022 [15]. Briefly, iHBs were seeded in agarose microwells, thus allowing their rearrangement and self-assembly within 24 h into well-defined spherical aggregates with a diameter of 200 µm ± 17 µm (*n* = 40). Approximately 1200 aggregates were then collected from the microwells and suspended per mL of 1.5% (*w*/*v*) alginate to form beads. The iHBs were further differentiated into iHeps within the beads until Day 28 (16 days after encapsulation) allowing self-organization of the aggregates into organoids (E-iHep-Orgs). Figure 2A illustrates the experimental scheme with a representative image of the aggregates thus formed. Morphological analysis of the resulting beads showed that the encapsulation system generated spherical beads with an average diameter of 1140 ± 38 µm (measured from images of 60 beads). The distribution of aggregates within the beads was not homogeneous; indeed, 41% beads contained one iHep-Org, and 17% and 12% contained two or three iHep-Orgs, respectively, while about 30% of beads were empty (*n* = 60). Under E-iHep-Org conditions, the Orgs remained entrapped within the alginate beads and retained their initial diameter (200 ± 17 µm) until the end of the culture on Day 28 (16 days post-encapsulation). In parallel, the non-encapsulated iHep-Orgs maintained their shape, compactness and diameter throughout the differentiation period up to 28 days in culture (Supplementary Materials Figure S3A). The viability assessment of E-iHep-Orgs over two weeks of encapsulation demonstrated high viability and very few dead cells (Figure 2B). These results highlighted the fact that encapsulating aggregates rather than single cells enabled the maintenance of cell viability within alginate beads.

To further investigate whether this approach might impact the differentiation process, we evaluated gene expression using RT-PCR (Figure 2C). The profile revealed the expression of hepatocyte marker genes (ALB, HNF4α, AFP, CYP3A4 and CYP3A7) for E-iHep-Orgs as well as under both 2D and iHep-Orgs control conditions, thus indicating the successful differentiation of iHBs into hepatocytes (iHeps). Interestingly, the gene expression of both AFP and CYP3A7 (the immature hepatocyte markers) had disappeared at Day 26 of differentiation (Day 14 post-encapsulation) under both 3D culture conditions (E-iHep-Orgs and iHep-Orgs) while these genes were still expressed under 2D conditions. Moreover, at Day 28, in line with the RT-PCR results, immunofluorescence analysis of E-iHep-Orgs and iHep-Orgs cryosections carried out up to their center revealed the homogenous distribution of mature hepatocyte markers such as ALB, HNF4α, CYP3A4, UGT1A1, CK8 and A1AT (Figure 2D and Supplementary Materials Figure S3B). This positive labeling, combined with the residual labeling or absence of AFP, confirmed that the iHeps differentiated within E-iHep-Orgs and iHep-Orgs had acquired a high level of maturation. Furthermore, the biliary markers CK7 and SOX9 were not expressed, showing the homogeneous differentiation of iHBs into iHeps. These results indicate that aggregation did indeed play an essential role in the differentiation of iHBs into hepatocytes and in their maturation and that encapsulation did not affect their differentiation either positively or negatively.

### 3.3. Functional Assessment of E-iHep-Orgs

**Figure 3:**
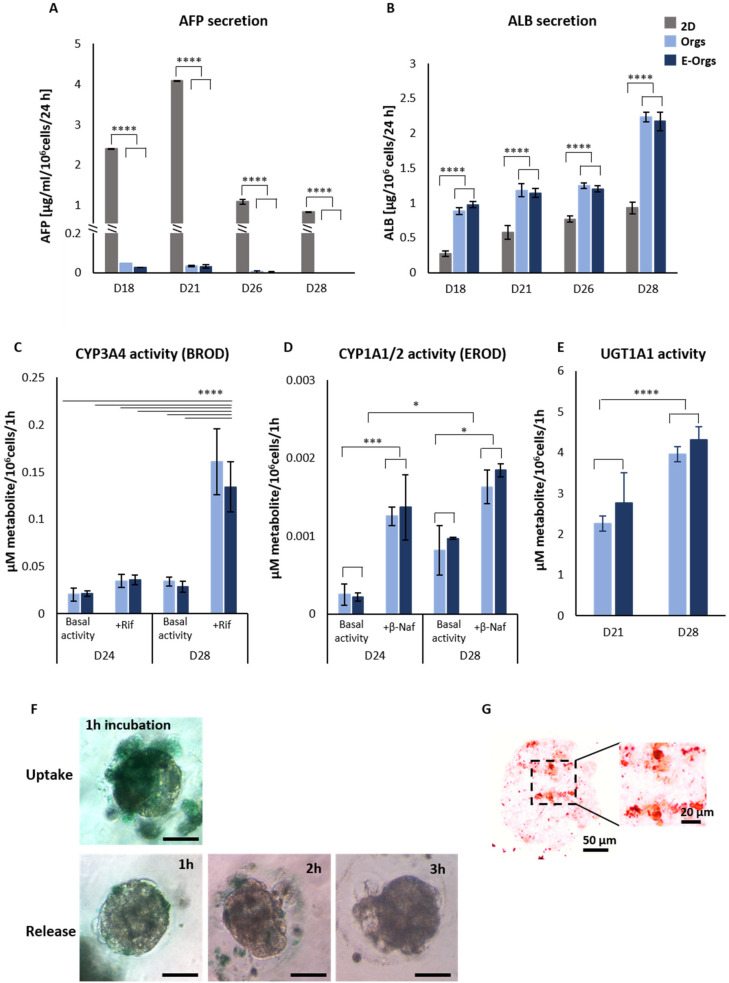
Functional assessment of iHep-Orgs and E-iHep-Orgs. (**A**,**B**) Plasma protein AFP and ALB secretion by iHeps at the indicated time points are shown. Histograms represent mean ± SD (*n* > 10). (**C**–**E**) Xenobiotic metabolism Phases I and II. CYP3A4 activity determined by BROD, histograms represent mean ± SD (*n* = 5); CYP1A1/2 activity determined by the EROD test. The histograms represent mean ± SD (*n* = 4). UGT1A1 activity; the histograms represent mean ± SD (*n* = 8). (**F**) Indocynanine green uptake and release assay in E-iHep-Orgs (scale bar: 100 µm). (**G**) Oil Red O staining of lipid droplets in E-iHep-Orgs. *p* < 0.0001 (****), *p* < 0.001 (***), and *p* < 0.05 (*).

Samples of the media were collected at different time points in order to quantify secreted α-fetoprotein (AFP) and albumin (ALB). AFP secretion progressively decreased until it was not detected anymore at Day 26 under the 3D culture conditions (E-iHep-Orgs and iHep-Orgs), whereas iHeps continued to secrete AFP under conventional 2D culture conditions (Figure 3A). The disappearance of AFP secretion in 3D culture conditions was associated with stable ALB secretion throughout the observed period (Days 18 to 26), with an average of about 1.2 µg/24 h/10^6^ cells. On Day 28, 2.2 µg/24 h/10^6^ cells of secreted albumin were detected, almost 2-fold higher than in 2D conditions (Figure 3B).

As mature functional hepatocytes play an essential role in the biotransformation of many xenobiotics and in drug metabolism, we assessed Phase I and II enzyme metabolisms in the E-iHep-Orgs and the iHep-Orgs (Figure 3C–E). During Phase I, CYP3A4 and CYP1A1/2 activities were measured based on EROD and BROD tests, respectively. As expected, E-iHep-Orgs and iHep-Orgs displayed basal activities of both CYP3A4 and CYP1A1/2 at Day 24 of differentiation. The basal level of CYP1A1/2 was significantly increased between Day 24 and Day 28. Following induction using rifampicin (as a CYP3A4 inducer) and β-naphthoflavone (as a CYP1A1/2 inducer), the CYP3A4 and CYP1A1/2 activities were induced 4-fold and 2-fold, respectively. During Phase II, the potential of E-iHep-Orgs and iHep-Orgs to conjugate the metabolite (4-MU) via the UGT1A1 enzyme was evaluated. UGT1A1 enzyme activity was detected at Day 21 of culturing and the level rose until Day 28. In addition, E-iHep-Orgs at Day 28 were able to uptake ICG after one hour of incubation and completely release the dye in less than three hours (Figure 3F), evidencing the presence of the functional transporters OATP1B3, NTCP, and MDR3. The ability of E-iHep-Orgs to store lipids was evidenced by Oil Red O staining of the lipid droplets in cells, as shown in Figure 3G. These findings indicate that E-iHeps had reached a significant level of maturation and functionality.

### 3.4. Functional Assessment of E-iHeps under Pathological Conditions

**Figure 4:**
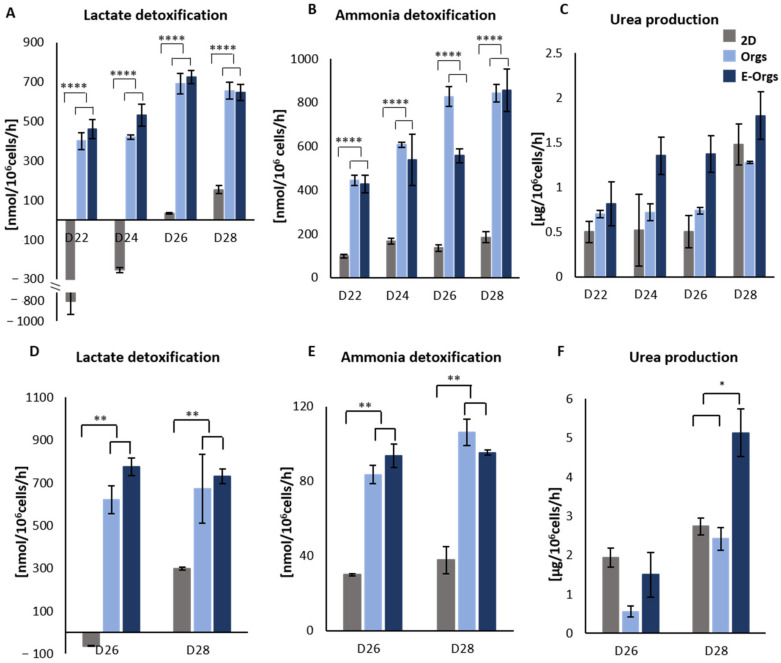
Detoxification abilities and urea production of iHeps. (**A**–**C**) Lactate and ammonia detoxification and urea production under moderate pathological conditions, respectively. Histograms represent mean ± SD (*n* > 10). (**D**–**F**) Lactate and ammonia detoxification and urea production under ultra-pathological conditions, respectively. Histograms represent mean ± SD (*n* > 5). *p* < 0.0001 (****), *p* < 0.01 (**) and *p* < 0.05 (*).

Finally, regarding their potential use in a clinical application to treat liver failure, we investigated the ability of iHeps to detoxify the typical toxins that accumulate in patients with acute liver failure (Figure 4). Thus, to evaluate their potential to remove lactate and ammonia, iHeps were incubated for 2 h with a medium containing 2 mM lactate and 1.5 mM ammonia (Figure 4A–C). We compared their detoxification ability at different time points. By Day 22, E-iHep-Orgs were able to detoxify both lactate and ammonia. These detoxification activities were enhanced by about 2-fold between Days 24 and 26, reaching a maximum level on Day 26 for lactate and ammonia with an average of 780 nmol/h/10^6^ cells and 800 nmol/h/10^6^ cells, respectively. The detoxification profiles of lactate and ammonia did not differ significantly between E-iHep-Orgs and iHep-Orgs, whereas they were significantly different compared to 2D culture condition. In contrast to the E-iHep-Orgs and iHep-Orgs, the 2D iHeps displayed observable lactate production until Day 24. Urea production was measured after treating the cells with 1.5 mM NH_4_Cl. Under the 3D culture conditions (E-iHep-Orgs and iHep-Orgs), iHeps were able to produce urea, suggesting ammonia elimination through ureagenesis. These results highlighted the ability of cells to detoxify lactate and ammonia supplemented in the culture. Therefore, the potential of iHeps to detoxify lactate and ammonia in a context of acute liver failure was investigated by mimicking plasma viscosity through the addition of 70 g/L BSA and higher lactate and ammonia concentrations of 7 mM and 2 mM, respectively, as the equivalent of an ultra-pathological state (Figure 4D,E). Similar to the high rate of ammonia and lactate detoxification observed with the previous moderate concentrations, only iHeps from Days 26 and 28 were subjected to equivalent ultra-pathological conditions. Again, lactate and ammonia detoxification activities were higher in iHeps under 3D culture conditions compared to conventional 2D conditions, by 4-fold and 5-fold, respectively.

Overall, our results showed that E-iHep-Orgs did not only exhibit the markers of mature hepatocytes but were also metabolically active.

## 4. Discussion

Due to the insufficient availability of PHHs and the problems encountered in maintaining their functionality in culture, the generation of functional human hepatocytes by differentiating hPSCs in vitro is of special significance in basic research and pathophysiological studies, as it is important for cell therapy applications and the development of extracorporeal bioartificial liver devices. One of the most important factors determining the success of engineered liver tissue is indeed the functionality of cells. Knowing that the maturation and functionality of hiPSC-derived hepatocytes can be achieved under a 3D configuration [15,16], the aim of this work was to demonstrate that differentiating iPSC-derived hepatocytes after alginate encapsulation is effective so that this renewable liver cell source could be confirmed. Some studies indicate that alginate and the encapsulation technique could be used to provide a mechanical support for 3D culture, which would assist cell functions [31,32]. Alginate encapsulation also provides a biocompatible biomaterial that enables mechanical protection, cryopreservation and simple handling [33]. We have thus described a combination of iPSC differentiation hepatocytes and alginate microencapsulation and have investigated the effects of microencapsulation on the cells, insofar as this approach is still under examination.

Several research groups have encapsulated pluripotent stem cells and investigated hepatocyte differentiation, though their analysis of liver functions has been limited [34,35,36,37]. Recently, Syanda et al., evaluated the encapsulation of hESC-derived hepatocyte aggregates in modified alginate and demonstrated the advantages of using this approach for transplantation in a mouse model [26]. However, they did not explore the effects of alginate encapsulation on the maturity and functionality of the hepatocytes thus differentiated. Xie et al., compared the encapsulation stages of hESCs in order to optimize their hepatic differentiation in alginate beads. They showed that encapsulating hESC-derived definitive endoderm (DE) was more effective than encapsulating hESCs in terms of the degree of differentiation and viability of the hepatocytes produced in alginate [37].

In parallel with these recently published studies and based on our experience of the hepatocyte differentiation of hPSCs and alginate encapsulation, we have attempted to validate such an approach using hepatoblasts differentiated from hiPSCs and to further test hepatocyte function in vitro. In order to demonstrate that alginate microencapsulation as a 3D microenvironment hydrogel-based approach was suitable for the differentiation of hiPSC-derived hepatoblasts (iHBs) into functional hepatocytes, we compared this system with conventional 2D conditions and with the scaffold-free 3D culture of aggregates. To optimize the homogeneity of the resulting population, we chose to encapsulate the cells at the iHB stage. Unexpectedly, and unlike the encapsulation of hESC-derived DE described by Xie et al. [37], iHBs encapsulated as single cells displayed a very low viability rate. Therefore, the alginate encapsulation of iHBs as single cells failed to provide a microenvironment that supported cell viability. We then combined the cell aggregation of iHBs as a 3D culture condition (as recently described by Messina et al. [15]) and followed it with alginate encapsulation. Our results have demonstrated that the encapsulation of preformed aggregates preserved viability and even enabled hepatocyte differentiation in a highly homogeneous manner. Indeed, hepatoblasts differentiated in alginate beads (E-iHep-Orgs) demonstrated successful hepatocyte maturation, as indicated by the disappearance of AFP secretion, and improved their characteristics during the period of culturing, such as the secretion of albumin and the detoxification of toxins and xenobiotics. Furthermore, we found that E-iHep-Orgs were both functional and mature in alginate beads, as were the non-encapsulated iHep-Orgs serving as controls. These results showed that alginate did not affect maturation or function of hepatocytes in iHep-Orgs. Therefore, a combination of aggregation and encapsulation technologies can be used to produce hepatocyte biomass, which when protected by encapsulation is suitable as a biological component in the bioreactor.

Although it is not easy to compare the functions of hepatocytes among different culture protocols, Table S5 demonstrates that E-iHeps were highly functional and mature with a potential to procure curative effects in the treatment of liver failure. The average functional levels of E-iHep-Orgs at Day 28 were higher than those obtained with liver cell lines and therefore closer to primary hepatocytes. Pasqua et al., examined the liver functions of HepaRG cell lines entrapped in alginate beads as single cells, this being the most efficient hepatocyte cell line [21]. They reported that HepaRG^TM^ were able to self-aggregate in alginate beads and also to perform liver functions such as ammonia and lactate detoxification and biotransformation activity. Although HepaRG^TM^ exhibit the most similar liver functions to PHHs, the hepatic activities of our E-iHep-Orgs were more substantial (see Table S5) and thus closer to PHHs. Xie et al., outlined that although it was promising, their approach was not efficient enough to generate a homogeneous and fully mature hepatocyte population [37]. Their encapsulated derived hepatocytes still expressed AFP, a marker of immature hepatocytes. By contrast, in our E-iHep-Orgs, AFP decreased and then disappeared over time, thus demonstrating a high degree of maturity. We also estimate that our culture system based on the encapsulation of hepatoblasts, the progenitors of hepatocytes, ensured better homogeneity, whereas the encapsulation of hESCs or definitive endoderm results in an immature and heterogeneous population. The degree of homogeneity found in our culture has never previously been recorded, which makes our approach more appropriate for further investigations and applications where functionality is of prime importance.

The strength of our approach lies in the combination of generating aggregates with uniform size and the alginate microencapsulation technology. During this study, pure alginate was used for encapsulation, but, in the future, hybrid hydrogels composed of alginate and extracellular matrix components (ECM), or even modified alginate, could also be used to mimic the in vivo microenvironment [38,39]. Furthermore, it is possible to foresee the use of multicellular aggregates that mimic the composition of the liver by mixing hepatocytes with other liver-specific cell types including cholangiocytes, endothelial cells or stellate cells [40,41]. The various hepatic functions displayed by our E-iHep-Orgs could meet the requirements to act as alternative cell sources to replace PHH in preclinical studies, including biological components for BAL and transplantation. As a very large quantity of biomass will be required for these applications, a scalable aggregation system will be needed in the future; currently, our aggregate formation technique is not suitable for truly large-scale production. In this context, aggregation systems using a 3D suspension system or a rotating bioreactor represent promising methods for mass production [42,43].

## 5. Conclusions

We have created a 3D differentiation method that consists in the stepwise monolayer differentiation of hiPSCs into hepatoblasts followed by a process of self-aggregation of the cells into aggregates for encapsulation. These culture conditions use non-adherent agarose microwells and a biocompatible biomaterial to produce differentiated hepatocytes encapsulated under 3D culture conditions from hiPSCs. The resulting E-iHep-Orgs express hepatocyte markers and display functional maturity and metabolic activity. The production of human functional hepatocytes from hiPSCs will be of considerable benefit for hepatocyte transplantation, BAL support devices and drug screening.

## Data Availability

Not applicable.

## Supplementary Materials

The following supporting information can be downloaded at: https://www.mdpi.com/article/10.3390/cells12060865/s1, **Materials and Methods:** Culture of human induced pluripotent stem cells, hiPSC differentiation into hepatoblasts (iHBs), Characterization of bead size and cell aggregation, Cell recovery from beads, Nucleic acid extraction, RT-PCR, Immunofluorescence staining. **Supplemental Figures:** Figure S1. Assessment of hiPSC differentiation into iHBs, Figure S2. Schematic representation of the alginate encapsulation procedure, Figure S3. Differentiated non-encapsulated iHeps (Organoids (Orgs)). **Supplemental Tables:** Table S1. Protocol for hiPSC differentiation into hepatoblasts, Table S2. Protocol for hepatoblast differentiation into hepatocytes, Table S3. List of primary antibodies used for immunofluorescence staining, Table S4. List of primer sequences used for gene expression analysis, Table S5. Summary table of hepatic functions. References [15,20,21,34,35,44,45,46] are cited in Supplementary Materials.

## Abbreviations

4-MU: 7-hydroxy-4-methylcoumarin; A1AT: alpha 1-antitrypsin; AFP: α-fetoprotein; ALB: albumin; AG: agregate; β-Naf: β-naphthoflavone; BAL: extracorporeal bioartificial liver devices; BMP4: bone morphogenetic protein 4; BROD: Benzyloxyresorufin-O-dealkylase; CYP450: cytochrome P450; CK-8: cytokeratin 8; CK-19: cytokeratin 19; CYP1A1/2: cytochrome P450 1A1/2; CYP3A4: cytochrome P450 3A4; CYP3A7: cytochrome P450 3A7; DE: definitive endoderm; Dex: dexamethasone; E-iHeps: encapsulated differentiated hepatocytes; E-iHep-Orgs: Encapsulated iHep organoids EROD: 7-ethoxyresorufin-O-deethylase; FDA: fluorescein diacetate; FGF2: fibroblast growth factor 2; FGF4: fibroblast growth factor 4; HCM: hepatocyte complete medium; HGF: hepatocyte growth factor; hPSCs: human pluripotent stem cells; hESCs: human embryonic stem cells; hiPSCs: human induced pluripotent stem cells; HNF4α: hepatic nuclear factor 4 α; ICG: indocyanine green; iHBs: hepatoblasts; iHeps: human iPSC-derived hepatocytes; MDR3: multidrug resistance associated protein 3; NTCP: Na+-taurocholate co-transporting polypeptide; OATP1B3: organic anion-transporting polypeptide 1B3; OSM: oncostatin M; OLT: orthotopic liver transplantation; PI: propidium iodide; PHHs: Primary human hepatocytes; Rif: rifampicin; RT-PCR: real-time polymerase chain reaction; SSEA4: stage-specific embryonic antigen-4; UGT1A1: Uridine diphospho- Glucuronosyltransferases 1A1; ZO-1: zona-occludens 1.
