# Supplementary material for: Microencapsulated Hepatocytes Differentiated from Human Induced Pluripotent Stem Cells: Optimizing 3D Culture for Tissue Engineering Applications
Source: Cells. 2023 Mar 10;12(6):865. doi: 10.3390/cells12060865 (PMC10047414; doi:10.3390/cells12060865)
Supplement: Supplementary file 1 [file cells-12-00865-s001.zip › cells-2161523-supplementary.pdf]

cells

Supplementary

Microencapsulated Hepatocytes Differentiated from Human Induced Pluripotent Stem Cells: Optimizing 3D Culture for Tissue Engineering Applications

Marwa Hussein [1,2], Mattia Pasqua [2,3], Ulysse Pereira [2,3], Nassima Benzoubir [1,2], Jean-Charles Duclos-Vallée [1,2], Anne Dubart-Kupperschmitt [1,2], Cecile Legallais [2,3,*,†] and Antonietta Messina [1,2,*,†]

1 UMR_S 1193, INSERM/Université Paris Saclay, F-94800 Villejuif, France
2 Fédération Hospitalo-Universitaire (FHU) Hépatinov, F-94800 Villejuif, France
3 UMR CNRS 7338 Biomechanics & Bioengineering, Université de Technologie de Compiègne, Sorbonne Universités, F-60203 Compiegne, France
* Correspondence: cecile.legallais@utc.fr (C.L.); antonietta.messina@inserm.fr (A.M.)
† These authors contributed equally to this work.

Supplemental Materials and Methods

- Human induced pluripotent stem cell culture
- hiPSC differentiation into hepatoblasts (iHBs)
- Characterization of bead size and cell aggregation
- Cell viability assay
- Cell recovery from beads
- Nucleic acids extraction
- RT-PCR
- Immunofluorescence staining

Supplemental Figures

- Figure S1. Assessment of hiPSC differentiation into iHBs
- Figure S2. Schematic representation of the alginate encapsulation procedure
- Figure S3. Differentiated non-encapsulated iHeps (Organoids (Orgs)

Supplemental Tables

- Table S1. Protocol for hiPSC differentiation into hepatoblasts
- Table S2. Protocol for hepatoblast differentiation into hepatocytes
- Table S3. List of primary antibodies used for immunofluorescence staining
- Table S4. List of primer sequences used for gene expression analysis.
- Table S5. Summary table of hepatic functions

Supplemental Materials and Methods

Culture of human induced pluripotent stem cells

The hiPSC line (A29) used in this study had previously been established by Steichen et al. in 2014[1]. Undifferentiated hiPSC colonies were grown on Geltrex™ LDEV-Free Reduced Growth Factor Basement Membrane Matrix (Gibco) coated plates and maintained in StemMACS™ iPS-Brew XF (Miltenyi Biotec) at 37°C, under 5% O_2 and 5% CO_2. The culture medium was refreshed every second day and the hiPSC colonies were routinely mechanically passaged for amplification at a ratio of 1:6 every week.

hiPSC differentiation into hepatoblasts (iHBs)

Hepatoblast differentiation was performed using our previously published protocol with slight modifications[2]. Briefly, hiPSC colonies were dissociated using a 0.5% Trypsin-EDTA solution (Gibco) and seeded at a cell density of $4x10^4$ cells/cm^2 with 10 mM Y-27632 on 0.1% porcine gelatin (Sigma-Aldrich) coated culture plates in StemMACS™ iPS-Brew XF. The hiPSCs were maintained at 37°C under 5% CO2 until 70% confluency, then the differentiation protocol was initiated using RPMI-1640 medium (Gibco) complemented with B-27 serum-free supplement (Life Technologies), 1X MEM non-essential amino acid solution (NEAA, Gibco), 1X glutamin (Gibco) and 1X penicillin-streptomycin (Gibco) supplemented with growth factors and cytokines. For definitive endoderm differentiation, the hiPSCs were treated with 5 nM CHIR99021 (Miltenyi Biotech) for one day and 100 ng/ml Activin A (Miltenyi Biotech) and 10 nM LY294002 (Sigma-Aldrich) for the next four days. To achieve hepatic specification, medium was then supplied with 50 ng/ml Activin A, 10 ng/mL BMP4 (R&D Systems) and 20 ng/mL FGF2 (Miltenyi Biotech) from day 5 to day 7 of differentiation. The hepatic endoderm cells thus obtained were treated with 20 ng/ml HGF (Miltenyi Biotech) and 30 ng/ml FGF4 (Miltenyi Biotech) for 2 days to generate hepatoblasts (iHBs).

Characterization of bead size and cell aggregation

The evolution of the size of beads and encapsulated aggregates over time was determined by measuring their diameter using open-source ImageJ software. For each time point, more than 40 phase contrast images are taken using the inverted microscope and used to measure the size of aggregates and beads. Means and standard deviations were calculated.

Cell recovery from beads

Cells were retrieved from the capsules by incubating with a depolymerizing solution (55 mM sodium citrate, 50 mM EDTA and 10 mM HEPES in PBS) for 5 min at 37°C. The cells were then collected by centrifugation at 480g for 10 minutes. The cell pellet was washed 3 times with PBS and the samples were processed immediately for nucleic acid extraction, or were frozen at -80°C.

Nucleic acid extraction

Total RNA and DNA were extracted using Trizol reagent (Sigma-Aldrich) and purified with the Direct-zol™ DNA/RNA MiniPrep extraction kit (Zymo Research) according to the manufacturer's instructions. The concentrations of extracted RNA and DNA were determined by Nanodrop UV-visible Lite (ThermoFisher). The DNA concentration was used to define the number of cells per sample and to normalize the metabolic activity of the cells, while the RNA concentration was used for gene expression analysis by RT-PCR.

RT-PCR

RT-PCR (reverse transcriptase-PCR) was executed using SuperScript™ First-Strand Synthesis System for RT-PCR (Invitrogen) with oligoDT primers, Platinium Taq DNA Polymerase (Invitrogen) and gene primers (Table S2), according to the manufacturer's instructions.

Immunofluorescence staining

Samples were fixed in 4% w/v paraformaldehyde (PFA) for 30 minutes and 4 hours, respectively. The aggregates and encapsulated aggregates were embedded in sucrose-gelatin solution and frozen in liquid nitrogen. The resulting blocks were cut into 7 μm thick sections on a Leica Biosystems Cryostat. The sections and 2D cells were then permeabilized with 0.1% v/v Triton X-100 in PBS for 20 minutes, and saturated with 3% w/v BSA (Sigma) for 30 minutes. The samples were incubated with primary antibodies overnight at 4°C. They were then rinsed 3 times with PBS, and incubated with the appropriate secondary antibodies for 1 hour at room temperature followed by DAPI incubation to counterstain the nuclei. The list of antibodies is shown in Table S3. The samples were mounted with mounting solution (DAKO) and coverslips, and images were acquired using the EVOS™ FL automated imaging system and/or a Leica SP5 confocal microscope.

Supplemental Materials and Methods

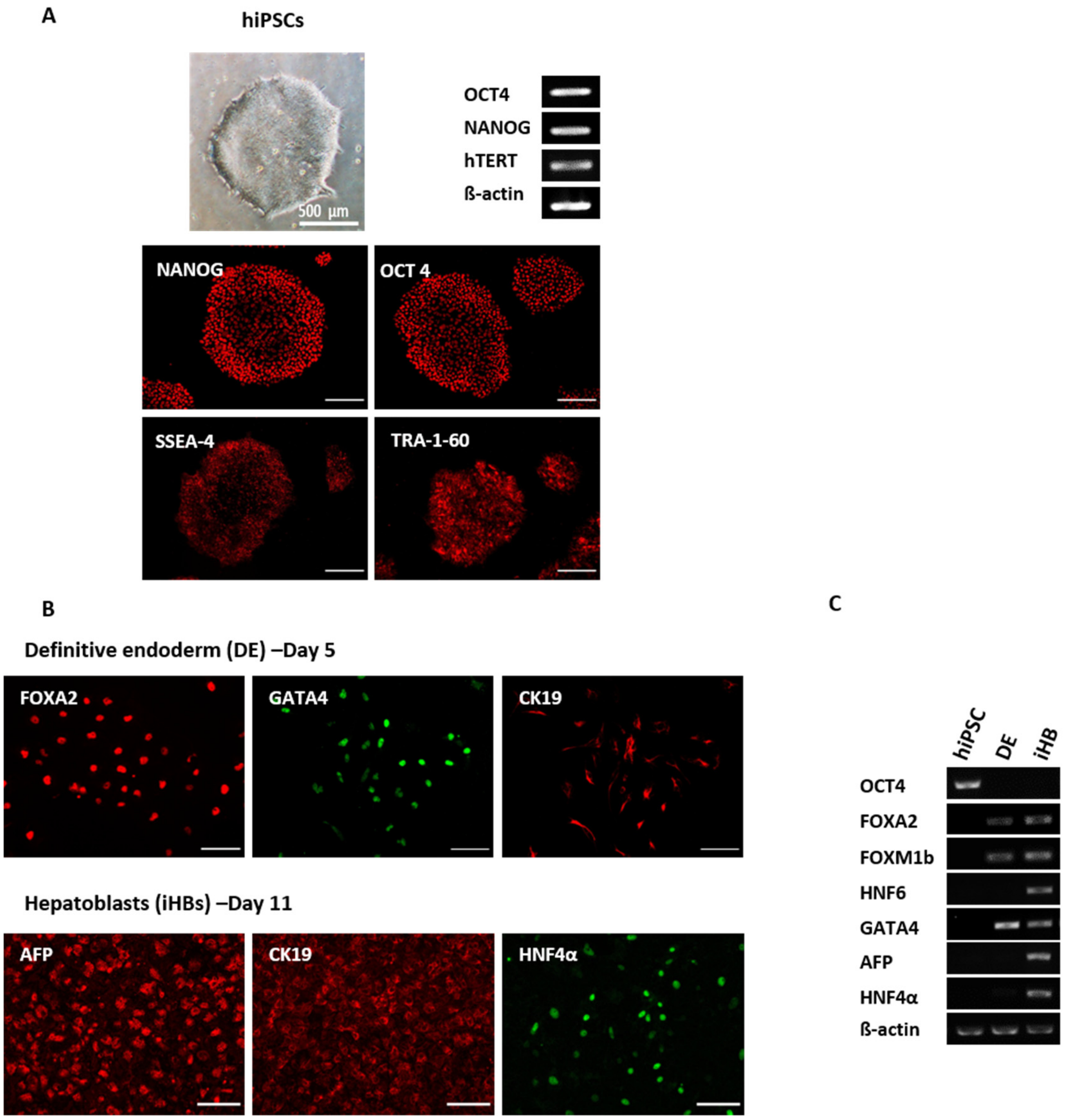

Figure S1. Assessment of hiPSC differentiation into iHBs. (A) Brightfield image and immunofluorescence staining of stemness markers of hiPSCs. (Scale bar= 200 µm). (B) Immunofluorescence labeling of endoderm and hepatoblast markers at day 5 and day 11, respectively. Scale bar = 100 µm. (C) RT-PCR analysis of the gene expression of stemness and hepatoblast markers over time for hiPSCs, the definitive endoderm (DE) and iHBs.

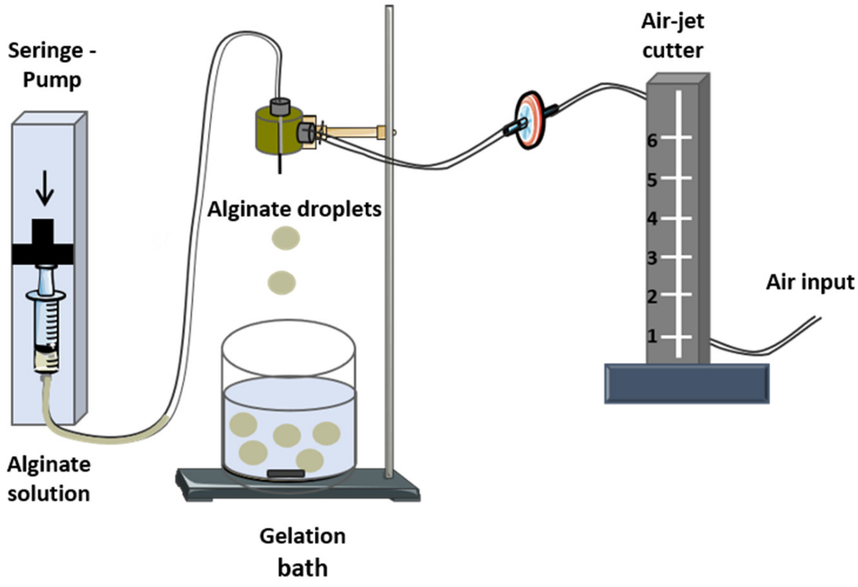

Figure S2. Schematic representation of the alginate encapsulation procedure.

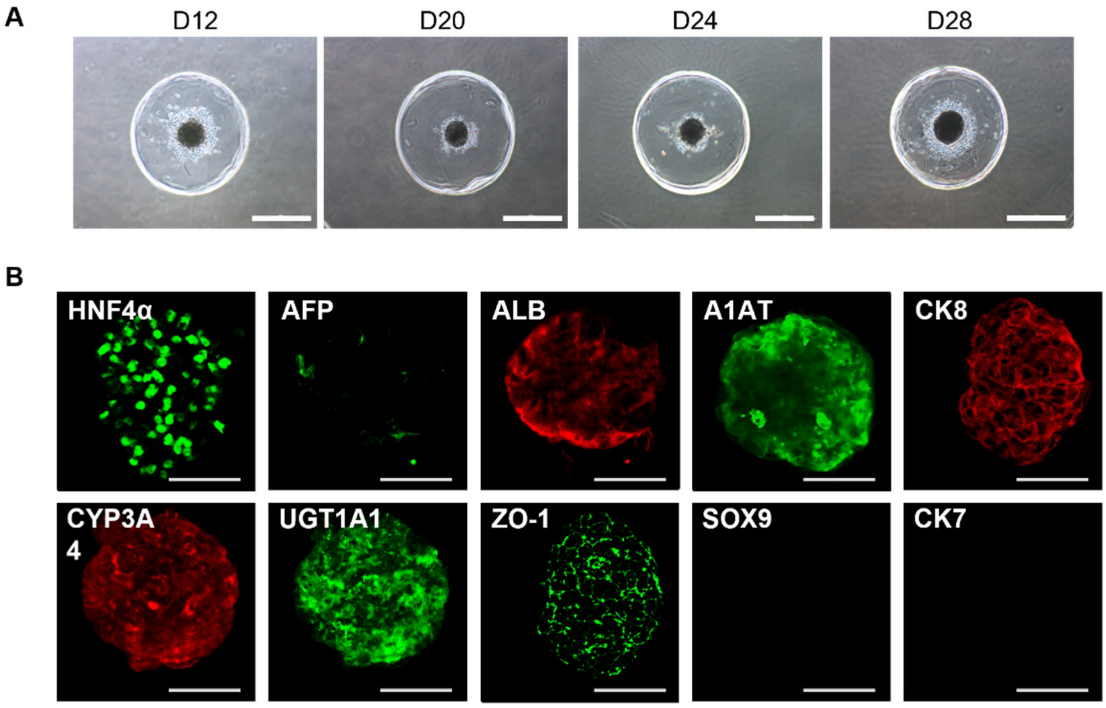

Figure S3. Differentiated non-encapsulated iHeps (Organoids (Orgs)). (A) Brightfield images of iHep-Orgs within agarose microwells. Scale bars = 500 µm. (B) Immunostaining analysis of hepatic-specific genes at final steps of iHep-Orgs. Scale bar= 150 µm. (C, D) Plasma protein AFP and ALB secretion by iHeps in Orgs and 2D are shown at the time points indicated. Histograms represent mean ± SD (n >10).

Supplemental Tables

Table S1. Protocol for hiPSC differentiation into hepatoblasts

Day	Culture medium	Growth factors and cytokines
-2 et -1	StemMACS™ iPS-Brew XF (Miltenyi Biotec)	10 μM Y27632
0	RPMI-1640 (Gibco) / B-27 (Life Technologies)	5 nM CHIR
1 – 4	RPMI-1640 (Gibco) / B-27 (Life Technologies)	100 ng/mL Activin A + 10 nM LY294002
5 – 7	RPMI-1640 (Gibco) / B-27 (Life Technologies)	10 ng/mL BMP4 + 20 ng/mL FGF2 + 50 ng/mL Activin A
8 – 9	RPMI-1640^{-Met} (Gibco)/ B-27 (Life Technologies)	20 ng/mL HGF + 30 ng/mL FGF4
10	HCM™ (Lonza)	20 ng/mL HGF + 10 ng/mL OSM +0.1 nM Dex

Table S2. Protocol for hepatoblast differentiation into hepatocytes

Day	Culture medium	Growth factors and cytokines
11 – 17	HCM™ (Lonza)	20 ng/mL de HGF +0.1 nM Dex + 20 ng/mL OSM
18 – 20	HCM™ (Lonza)	20 ng/mL de HGF +0.1 nM Dex + 20 ng/mL OSM + 10 ng/mL VK
21	HCM™ (Lonza)	20 ng/mL de HGF +0.05 nM Dex + 20 ng/mL OSM + 10 ng/mL VK + 0.5 nM CE and 5 nM SB
22 - 23	HCM™ (Lonza)	20 ng/mL de HGF +0.1 nM Dex + 10 ng/mL OSM + 10 ng/mL VK + 0.5 nM CE and 5 nM SB
24 - 25	HCM™ (Lonza)	20 ng/mL de HGF +0.05 nM Dex + 5 ng/mL OSM + 10 ng/mL VK + 0.5 nM CE and 5 nM SB
26 - 27	HCM™ (Lonza)	20 ng/mL de HGF + 1 nM Dex + 10 ng/mL VK + 0.5 nM CE and 5 nM SB
28	HCM™ (Lonza)	20 ng/mL de HGF + 0.5 nM Dex + 10 ng/mL VK + 0.5 nM CE and 5 nM SB

Table S3. List of primary antibodies used for immunofluorescence staining

Protein	Reference	Type	Dilution
NANOG	R&D AF1997	Polyclonal rabbit IGG	1/100
TRA-1-60	Millipore MAB4360	Polyclonal mouse IGG	1/150
SSEA-4	Millipore MAB4304	Polyclonal mouse IGG	1/150
OCT3/4	Santa Cruz sc-5279	Monoclonal mouse IgG	1/200
GATA4	Santa Cruz sc-1237	Polyclonal goat IgG	1/200
HNF3β (FoxA2)	Santa Cruz sc-6554	Polyclonal goat IgG	1/100
HNF4α	Santa Cruz SC-8987	Polyclonal rabbit IGG	1/200
AAT (α-1-antitrypsine)	DAKO A0012	Polyclonal rabbit IGG	1/200
AFP	Santa Cruz SC-8399	Polyclonal rabbit IGG	1/200
ALB	DAKO A0001	Polyclonal rabbit IGG	1/200
	Sigma A6684	Monoclonal mouse IgG	1/200
CK7	DAKO M7018	Monoclonal mouse IgG	1/100
CK18	Santa Cruz sc-8020	Monoclonal mouse IgG	1/200
CK19	DAKO M0888	Monoclonal mouse IgG	1/200
CYP3A4	Santa Cruz sc-53850	Monoclonal mouse IgG	1/100
SOX9	Santa Cruz sc-166505 AF488	Monoclonal mouse IgG	1/100
UGTA1	Santa Cruz sc-271268	Monoclonal mouse IgG	1/200
ZO-1	Biotechne NBP1-85047	Polyclonal rabbit IgG	1/200
F-Actin (Phalloidin)	Life Technologies A12381		1/200

Table S4. List of primer sequences used for gene expression analysis

OCT4	GTG GAG GAA GCT GAC AAC AA
	CAG GTT TTC TTT CCC TAG CT
NANOG	TCC AAC ATC CTG AAC CTC AG
	GAC TGG ATG TTC TGG GTC TG
HNF3β/FOXA2	GCG ACC CCA AGA CCT ACA G
	GGT TCT GCC GGT AGA AGG G
HNF6	ATC CAC AAC TCC CAG CAA GG
	GGT GTG TTG CCT CTA TCC TT
AFP	TTT TGG GAC CCG AAC TTT CC
	CTC CTG GTA TCC TTT AGC AAC TCT
A1AT	CCA ACA GCA CCA ATA TCC ATC TTC
	GTC CTC TTC CTC GGT GTC CTT G
HNF4α	CGG GTG TCC ATA CGC ATC CTT G
	GAC CCT CCC AGC AGC ATC TCC T
ALB	CCT TTG GCA CAA TGA AGT GGG TAA CC
	CAG CAG TCA GCC ATT CAC CAT AGG
CYP3A4	GAT TTG GCT CCT CTG CTT CT
	GCC TCC TGT GTA GTG AGA TTA C
CYP3A7	ATT CCA AGC TAT GTT CTT CAT CAT
	AAT CTA CTT CCC CAG CAC TGA
ACTIN	GCA CTC TTC CAG CCT TCC TTC C
	CTG CTG TCA CCT TCA CCG TTC C

Table S5. Summary table of hepatic functions. This Table recapitulates the function profiles obtained with E-iHep-Orgs by comparison with those obtained from studies based on the encapsulation of human hepatocytes of different origins.

Type de cell	ALB synthesis [µg/10^6 cells/24h]	Urea synthesis [µg/ml/10^6 cells/h]	phase I [nmol/10^6 cells/1h]		Lactate detoxification [nmol/10^6 cells/h]	Ammonia detoxification [nmol/10^6 cells/h]
E-iHeps – Our experimental results	2.1	1.8± 0.26	CYP1A1/2	1.8	646 ± 41	857 ± 98
			CYP3A4	130 ± 26		
Encapsulated HepG2 cells[3]	54 ±8	-	CYP1A2 (MROD)	0.576 ± 0.036	-	-
			CYP3A4 (BROD)	2.35 ± 0.36		
Encapsulated C3A cells[4]	2	0.08	-	-	-	5.7
Encapsulated HepaRG cells[5]	30 ± 19	-	CYP1A2 (EROD)	0.00067± 0.00024	167±24	119±2
			CYP3A4 (BROD)	0.00148 ± 0.0003		
Encapsulated human fetal hepatoblasts [6]	16	0.25	-	-	-	-
Encapsulated human ESCs [7]	0.15	5	-	-	-	-
Encapsulated human ESC aggregates[8]	1.7	1.1	-	-	-	-

Supplemental references

1. Steichen, C.; Luce, E.; Maluenda, J.; Tosca, L.; Moreno-Gimeno, I.; Desterke, C.; Dianat, N.; Goulinet-Mainot, S.; Awan-Toor, S.; Burks, D.; et al. Messenger RNA- versus Retrovirus-Based Induced Pluripotent Stem Cell Reprogramming Strategies: Analysis of Genomic Integrity. *Stem Cells Transl. Med.* **2014**, *3*, 686–691, doi:10.5966/sctm.2013-0158.
2. Messina, A.; Luce, E.; Benzoubir, N.; Pasqua, M.; Pereira, U.; Humbert, L.; Eguether, T.; Rainteau, D.; Duclos-Vallée, J.-C.; Legallais, C.; et al. Evidence of Adult Features and Functions of Hepatocytes Differentiated from Human Induced Pluripotent Stem Cells and Self-Organized as Organoids. *Cells* **2022**, *11*, 537, doi:10.3390/cells11030537.
3. Rahman, T.M.; Selden, C.; Khalil, M.; Diakanov, I.; Hodgson, H.J.F. Alginate-Encapsulated Human Hepatoblastoma Cells in an Extracorporeal Perfusion System Improve Some Systemic Parameters of Liver Failure in a Xenogeneic Model. *Artif. Organs* **2004**, *28*, 476–482, doi:10.1111/j.1525-1594.2004.07259.x.
4. David, B.; Dufresne, M.; Nagel, M.-D.; Legallais, C. In Vitro Assessment of Encapsulated C3A Hepatocytes Functions in a Fluidized Bed Bioreactor. *Biotechnol. Prog.* **2004**, *20*, 1204–1212, doi:10.1021/bp034301z.
5. Pasqua, M.; Pereira, U.; Messina, A.; de Lartigue, C.; Vigneron, P.; Dubart-Kupperschmitt, A.; Legallais, C. HepaRG Self-Assembled Spheroids in Alginate Beads Meet the Clinical Needs for Bioartificial Liver. *Tissue Eng. Part A* **2020**, ten.tea.2019.0262, doi:10.1089/ten.tea.2019.0262.
6. Cheng, N.; Wauthier, E.; Reid, L.M. Mature Human Hepatocytes from Ex Vivo Differentiation of Alginate-Encapsulated Hepatoblasts. *Tissue Eng. Part A* **2008**, *14*, 1–7, doi:10.1089/ten.a.2007.0131.
7. Maguire, T.; Davidovich, A.; Wallenstein, E.; Novik, E.; Sharma, N.; Pedersen, H.; Androulakis, I.; Schloss, R.; Yarmush, M. Control of Hepatic Differentiation via Cellular Aggregation in an Alginate Microenvironment. *Biotechnol. Bioeng.* **2007**, *98*, 631–644, doi:10.1002/bit.21435.
8. Fang, S.; Qiu, Y.; Mao, L.; Shi, X.; Yu, D.; Ding, Y. Differentiation of Embryoid-Body Cells Derived from Embryonic Stem Cells into Hepatocytes in Alginate Microbeads in Vitro. *Acta Pharmacol. Sin.* **2007**, *28*, 1924–1930, doi:10.1111/j.1745-7254.2007.00713.x.
